# Differential expression of proliferation and immune response genes between children and adults influences survival of diffuse large B cell lymphoma

**DOI:** 10.1038/s41598-025-04349-x

**Published:** 2025-06-03

**Authors:** Tamara Mangiaterra, Ruth Alonso-Alonso, Andrés Rabinovich, David Herman, Marcela De Dios Soler, Laura Galluzzo, Marcela Soria, Sandra Colli, Elena De Matteo, Socorro María Rodriguez Pinilla, Paola Chabay

**Affiliations:** 1 Multidisciplinary Institute for Investigation in Pediatric Pathologies (IMIPP), CONICET-GCBA, Molecular Biology Laboratory, Pathology Division, Ricardo Gutierrez Children’s Hospital, Gallo 1330, C1425EFD Buenos Aires, Argentina; 2 Pathology Department, Hospital Universitario Fundación Jiménez Díaz, Madrid, Spain; 3 Ecole des hautes études en santé publique (EHESP), Paris, France; 4 Pathology Division, Marie Curie Hospital, Buenos Aires, Argentina; 5 Pathology Division, Prof. Dr. Juan P. Garrahan Hospital, Buenos Aires, Argentina; 6 Hematology Division, Ricardo Gutierrez Children’s Hospital, Buenos Aires, Argentina; 7 Pathology Division, Ricardo Gutierrez Children’s Hospital, Buenos Aires, Argentina

**Keywords:** DLBCL, EBV, Gene expression, Pathways, Survival, Children, Cancer microenvironment, Haematological cancer, Tumour biomarkers, Tumour immunology, Tumour virus infections

## Abstract

Diffuse large B-cell lymphoma (DLBCL) is a neoplasm affecting adults and children, with different clinical behaviors between age groups. To shed light on those differences, gene expression profiling was evaluated in 48 formalin-fixed paraffin-embedded biopsies of patients with DLBCL. Sixteen differentially expressed genes in pediatric DLBCL compared to adults were demonstrated, involving lymphocyte differentiation, oncogenic signaling and chemotaxis. Pathway analysis confirmed the enrichment in proliferation-related pathways. In addition, the increased presence of NKCD56dim cells in pediatric patients suggests a cytotoxic immune response in this group, perhaps explaining their better outcome. Exclusion of Epstein Barr virus (EBV) + DLBCL, NOS, showed, in pediatric cases, additional downregulated genes associated with immune regulators and checkpoint genes. This suggested EBV infection may have on the modulation of immune response in pediatric lymphomas. Survival analysis showed associations between genes such as *MYC*, *NT5E* and *CD34*, and event-free survival in pediatric patients. Furthermore, higher expression of *MYC* in children displayed higher risk of death or relapse, while lower expression of *NT5E* and *CD34* was associated with lower risk. This study identifies distinct immune response and proliferation gene expression patterns in pediatric DLBCL compared to adults, and the interaction with tumor microenvironment, with potential implications for disease pathogenesis.

**Supplementary Information:**

The online version contains supplementary material available at 10.1038/s41598-025-04349-x.

## Introduction

Diffuse large B-cell lymphoma (DLBCL) is a neoplasm of large B-lymphocytes that accounts for 25–30% of B-cell non-Hodgkin lymphomas. It is the most common lymphoma subtype in western countries and occurs more often in men than women (1.3:1). DLBCL can affect both children and adults, with a median age of 70 years. It is an aggressive lymphoma, with approximately 70% of patients exhibiting advanced disease (stage III-IV). However, with current treatments, approximately 60–65% of patients achieve a complete response^1^. In adults, the prognosis of DLBCL depends on its cell of origin. Therefore, in order to distinguish the subtypes with a profile of germinal center (GC) origin, with a better prognosis, from those with an activated or post-germinal center (post-GC) profile, the use of an algorithm combining three markers: CD10, Bcl-6 and MUM-1 detected by immunohistochemistry (IHC) have been proposed^2^.

Despite considerable progress in the understanding of the pathogenesis of adult DLBCL, data from the pediatric population are still limited. It has been suggested that there are differences between both groups in terms of cell of origin, genetic abnormalities and response to current treatments. A better prognosis has been described for pediatric patients. Both genetic and immunophenotypic features may explain the reason for these differences^3^.

The immune microenvironment plays an important role in the pathogenesis of DLBCL, is involved in tumor growth and treatment resistance, and the maturity of this immune microenvironment, like the immune system, is age-dependent^4,5^. Furthermore, the characteristic composition of the DLBCL microenvironment is associated with differential response to treatment, the reason why its characterization may have a great clinical impact^6,7^. One of the immune escape mechanisms described to promote lymphomagenesis in adults is the expression of regulatory and tolerogenic markers, such as PDL1, TIM3 and LAG3. TIM3 and LAG3 are expressed on the surface of CD4 and CD8 T cells, whereas PDL1 may be expressed in the tumor cell as well as in the microenvironment, and it induces tolerance when it binds to its ligand PD1 expressed on T cells. The expression of all these tolerogenic markers is associated with a poorer overall survival in adult DLBCL^8,9^.

Gene expression profiling (GEP) studies are used to quantitatively analyze the expression of genes involved in proliferation to trigger tumorigenesis, as well as other genes such as tumor microenvironment-related factors with prognostic and/or biological significance. GEP also has been used to classify different molecular subtypes of DLBCL, based on genes expressed by different markers observed at the germinal center or after germinal center maturation, in order to define the cell of origin (COO) in DLBCL. The COO was used to identify more specific prognostic models and improve diagnostic accuracy^9,10^. The expression of genes such as *MYC/BLC2* has been described as risk indicators for survival in adults with DLBCL. However, studies of the relationship between gene expression and survival are limited, in particular concerning pediatric patients^11^.

Epstein Barr virus (EBV) + DLBCL not otherwise specified (NOS), is a new entity confirmed by the World Health Organization (WHO) in 2017 and 2022^1,11^, and by the Clinical Advisory Committee^12^, with a high incidence in elderly patients. Both defined a positive case when at least 80% of the tumor cells are positive for viral transcript EBERs by in situ hybridization (ISH). However, other groups, like ours, reported 20% of EBV + tumor cells to define a case as EBV + DLBCL^13^, and revealed EBV presence in 9.3% and 26% in adult and children, respectively^14,15^. The virus may contribute to some changes in the tumor microenvironment, such as the dysregulation of PDL1, in this new entity^16^.

Until now, there are no reports on the differential expression of immune response and proliferation genes in pediatric DLBCL, which would be important for understanding the age-related differences in the pathogenesis of this disease. Therefore, the aim of this study is to evaluate the expression of immune response and proliferation genes in pediatric patients with DLBCL in comparison with adult patients, including EBV + DLBCL NOS cases, and their impact on event-free survival in pediatric patients.

## Results

### General characteristic of the DLCBL series

In order to assess gene expression profile in pediatric cases, 26 DLBCL cases were included in this study, and compared with 22 DLBCL adult cases. Of the total number of cases, 25 were male and 23 were female. Twenty-six cases (54%) were classified as non-GC subtype, 21 (44%) were classified as GC subtype, and the remaining, 1 case (2%), were unclassified, according to Hans’ IHC classification^2^. Supplementary table S1 shows the histological subtypes of each age group in detail. The age range was 2–16 in pediatric patients (median 9 years), while in adults age range was 19–73 (median 60 years).

### nCounter gene-expression assay

**Fig. 1:**
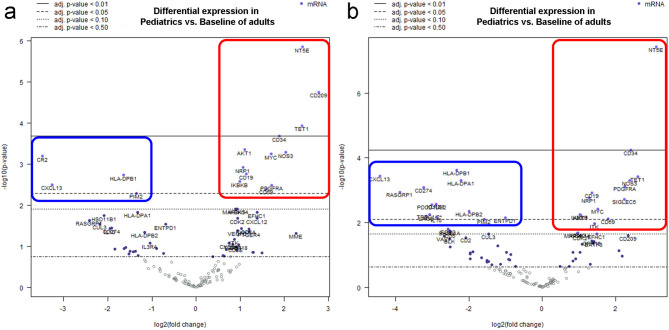
Volcano blots plot for each group analyzed. (**a**) The upregulated (red)/downregulated (blue) genes for Pediatrics vs. Adults DLCBL, NOS cases, including EBV + cases. (**b**) The upregulated (red)/downregulated (blue) genes for Pediatrics vs. Adults DLCBL cases excluding EBV + cases.

Immune response gene expression profiling, using a transcriptomic approach, was evaluated in 26 pediatric cases, and compared with 22 adult DLBCL cases. In a first assay, when DLBCL were compared between pediatric and adult, 16 differentially expressed genes were identified (FDR < 0.05 and log2 FC > 2, Fig. 1a). Differential expression analysis showed that 12 genes were significantly up-regulated and 4 down-regulated in DLCBL pediatric patients, compared to adult DLBCL. According to Genecard analysis, the top 4 genes with higher expression in pediatric DLBCL (FDR < 0.01 and log2 FC > 2, Fig. 1a) are involved in the differentiation of lymphocytes (*NT5E*), regulation of dendritic cell-induced T cell proliferation (*CD209*), T and B cell development and regulation of inflammation (*TET1*), and MHC class I and innate immune system-mediated antigen processing and presentation, in addition to their role in early hematopoiesis (*CD34*). The remaining 8 significantly upregulated genes in the pediatric cases (FDR < 0.05 and log2 FC > 2, Fig. 1a) are associated with the activation of signaling pathways, such as PI3L/ALK and NF-κb (*IKBKB*), which enables cell proliferation and survival (*ALK*). In addition, oncogenes important for proliferation in DLBCL (*MYC*) and associated with T cell proliferation and activation (*CD69*) were also found in this upregulated group, as well as a B-cell marker (*CD19*), a receptor kinase signaling (*NRP1*), an angiogenesis inductor (*NOS3*) and a tumor progression inducer (*PDGRFA*). Among the 4 significantly downregulated genes (FDR < 0.05 and log2 FC > 2, Fig. 1a) in pediatric patients, an oncogene (*PIM2*), a chemokine (*CXCL13*), an antigen-presenting molecule (*HLA-DPB1*) and a complement and Epstein-Barr virus receptor (*CR2*) are significantly downregulated. Supplementary Table S2 shows the top 20 genes analyzed in the whole cohort.

Even though the WHO described a cutoff of at least 80% of EBERs + cells to define EBV + DLBCL, our group previously described the influence of EBV in the pathogenesis of DLBCL in pediatric and adult patients, and defined EBV + DLBCL as cases with more than 20% of EBERs + cells. This cutoff was based on the observation that only cases above this threshold displayed latency II or III patterns, which are characteristic of EBV + DLBCL^13–15^. Of the 48 cases, 37 were EBV-negative and 11 EBV-positive (3 adult and 8 pediatric). Therefore, to evaluate exclusively in the 37 EBV-negative cases the expression of immune genes, a second analysis was carried out to compare pediatrics with adults DLBCL cases excluding EBV + DLBCL NOS cases. Twenty-five genes were identified as significant differentially expressed genes, 12 upregulated and 13 downregulated in pediatric DLCBL cases compared to adult ones, excluding EBV + DLCBL NOS cases (FDR < 0.05 and log_2_ FC > 2, Fig. 1b). The main differences were proved in differentially downregulated genes expressed in the pediatric cases, since most significantly upregulated genes in EBV-negative cases were similar to those upregulated in the whole series (Fig. 1b). In contrast, even though downregulation of *CR2*, *PIM2*, *CXCL13* and *HLA-DPB1* were demonstrated in all DLBCL and in EBV-negative DLBCL cases, 10 new genes were differentially downregulated. Those genes included the major histocompatibility complex–associated genes (*HLADPA1*,* HLADPB2*), T cells genes (*TRBC1/2*), important immunoregulators (*IL-10*), immune checkpoint genes (*LAG3* and *CD274*), as well as *RASGRP1*,* PDCD1LG-2* and *ENTPD1*. Supplementary Table S3 shows the top 20 genes analyzed. Remarkably, when only EBV + DLBCL cases were evaluated, no significant differences were observed in genes up and down regulated when children and adult cases were evaluated (Supplementary Fig. S1).

**Fig. 2:**
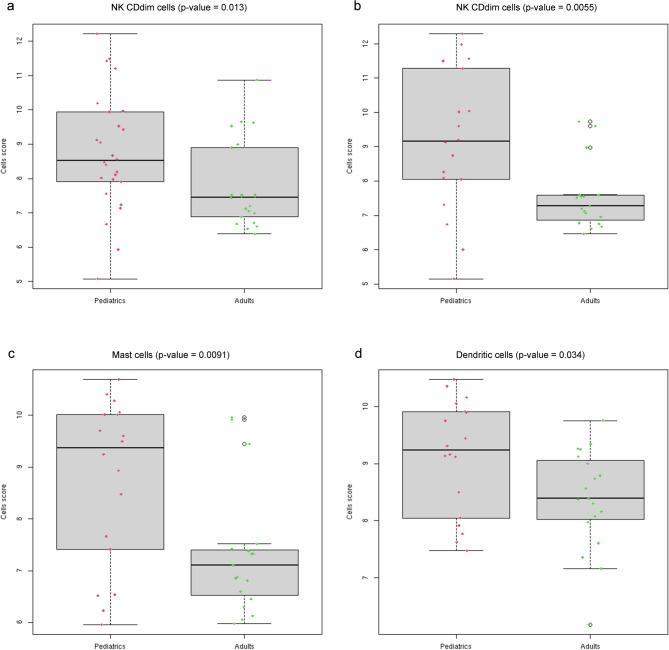
Box plot for each group analyzed. (**a**) NKCD56dim cells types between Pediatric vs. Adults DLCBL, NOS total cases. (**b**) NKCD56dim cells types between Pediatric vs. Adults DLCBL excluding EBV + cases. (**c**) Mast cells types between Pediatric vs. Adults DLCBL excluding EBV + cases. (**d**) Dendritic cells types between Pediatric vs. Adults DLCBL excluding EBV + cases.

The combination of immune cell response genes allows the identification of different cell types. In order to evaluate the cell types expressed in pediatric DLBCL compared to the adult ones, gene expression was analyzed for the different cell types presented in the gene panel. Only a significant increase of gene expression specific for NKCD56dim cells was observed in pediatric patients (*p* = 0.013, MW test, Fig. 2a). Concerning analysis for differential genes expressed in cell types, specifically in EBV-negative DLCBL cases, an increase of gene expression not only for NKCD56dim cells (*p* = 0.0055 MW test), but also for mast cells (*p* = 0.0091, MW test) and dendritic cells (*p* = 0.034, MW test) were observed in pediatrics cases compared with adult ones (Fig. 2b-d).

### Pathway enrichment analyses

**Fig. 3:**
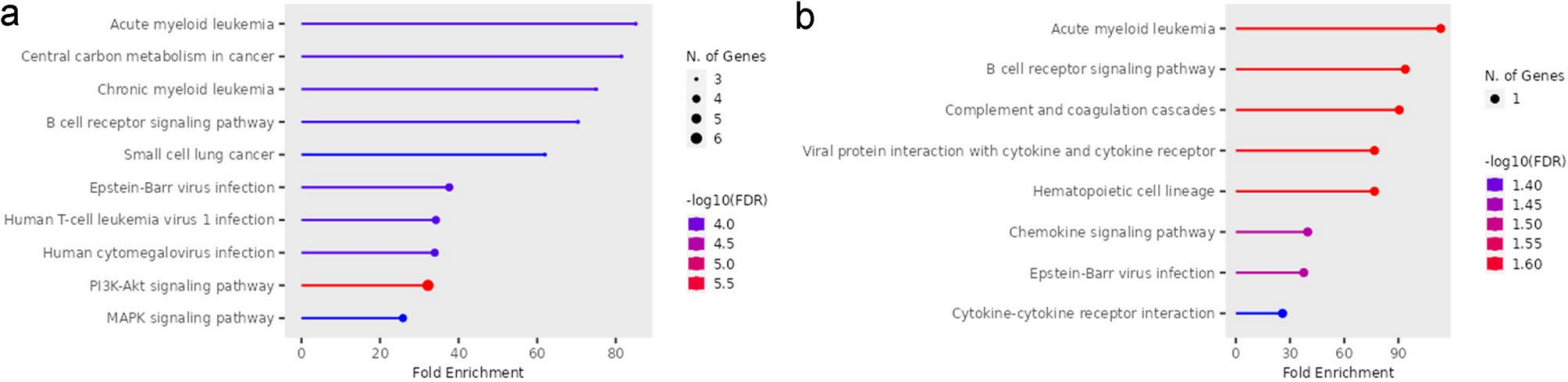
Diagram KEGG pathway enrichment analysis for DLCBL, NOS total cases^42^. (**a**) Pathway for significantly upregulated genes. (**b**) Pathway for significantly downregulated genes.

The KEGG pathways enrichment analysis is shown in Supplementary Tables S4 a-b for the whole DLCBL, NOS cases. In those cases, the analysis for both upregulated and downregulated genes displayed an enrichment in pathways involved in proliferation such as Acute myeloid leukemia, Central carbon metabolism in cancer, Chronic myeloid leukemia, B cell receptor signaling pathway, small cell lung cancer, PI3K-Akt signaling pathway, MAPK signaling pathway. Besides, Epstein-Barr virus infection, Human T-cell leukemia virus 1 infection and Human cytomegalovirus infection pathways were also enriched. As observed in Supplementary Table S4b, the PI3K-Akt and MAPK signaling pathways were the only ones that were not enriched for downregulated genes (Fig. 3a-b).

**Fig. 4:**
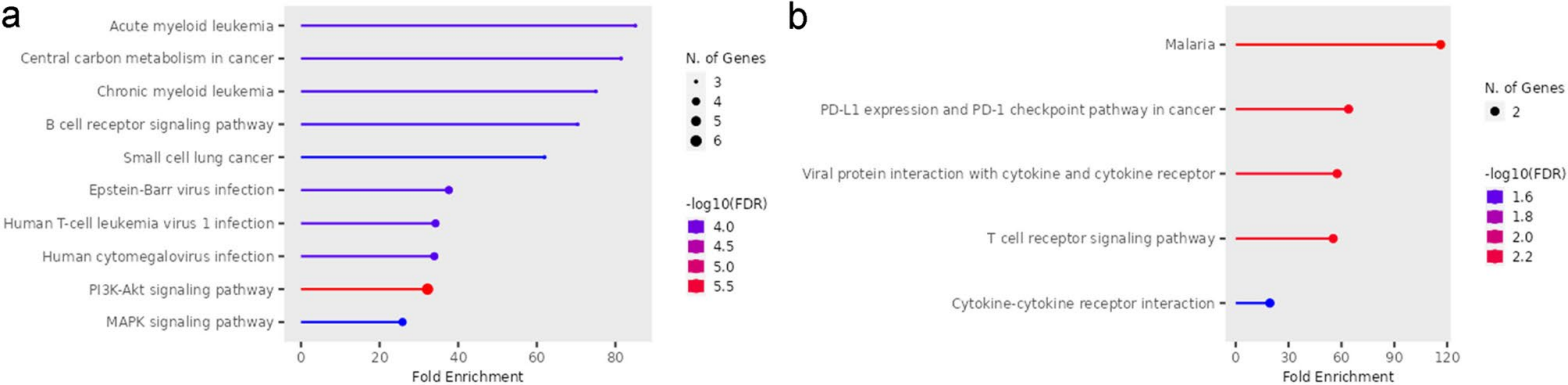
Diagram KEGG pathway enrichment analysis for DLCBL, NOS excluding EBV + cases^42^. (**a**) Pathway for significantly upregulated genes. (**b**) Pathway for significantly downregulated genes.

In the second group, since no important differences were observed in EBV + DLBCL cases, a second analysis was focused on EBV-negative DLBCL, NOS cases. Pathway enrichment analysis is detailed in Supplementary Tables S5 a-b for DLBCL excluding EBV + cases. Remarkably, the pathways enriched were different for upregulated and downregulated genes (Fig. 4a). For downregulated genes, significant enrichment was observed for PDL1 expression and PD1 checkpoint pathway in cancer, T cell receptor signaling pathway, viral protein interaction with cytokine and cytokine receptor, malaria, and cytokine-cytokine receptor interaction (Fig. 4b), whereas the pathways enriched for upregulated genes were similar the pathways affected for the entire DLBCL, NOS series.

### Survival

**Fig. 5:**
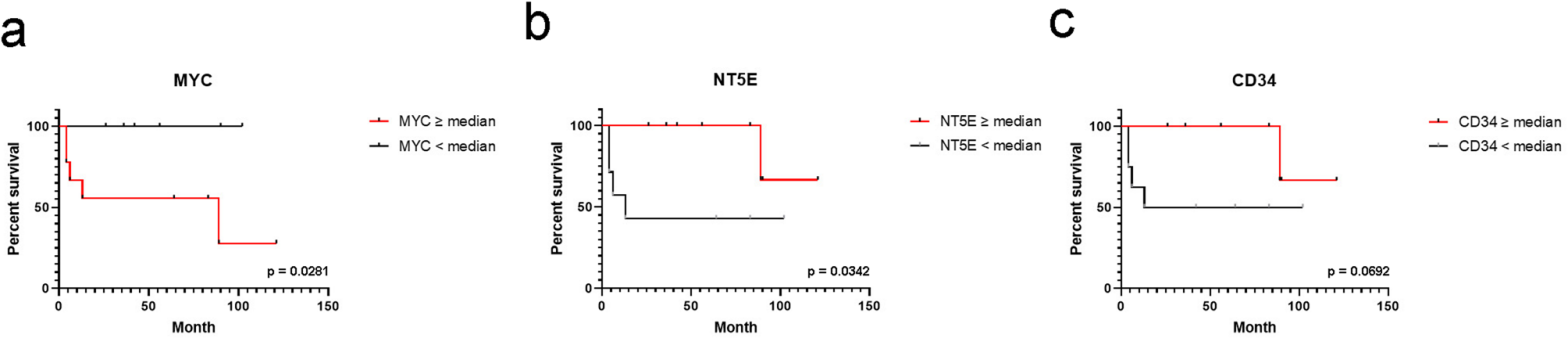
Event-free survival in pediatric patients with DLBCL, NOS. (**a**) Event-free survival for MYC gene. (**b**) Event-free survival for NT5E gene. (**c**) Event-free survival for CD34 gene.

As the expression of immune escape markers had been associated with survival in adult DLBCL, event-free survival was analyzed in pediatric patients^9^. Pediatric patients who expressed *MYC* genes above the median had lower event-free survival (*p* = 0.0281). Patients who express *NT5E* genes below the median had lower event-free survival (*p* = 0.0342,), while a trend was observed for *CD34* in the same scenario (*p* = 0.0692) (Fig. 5a-c).

For the univariable Cox regression analysis, patients with higher levels of *NT5E* and *CD34* gene expression presented a lower risk of death or relapse (*p* = 0.035, *p* = 0.049, respectively; Supplementary Table S6). Higher *M*YC expression was associated with an increased risk of events (*p* < 0.0001, Supplementary Table S6). A larger significant association was revealed for the mentioned genes when adjusting by age, sex, histological subtype and EBV status in the multivariable Cox model (*MYC* *p* < 0.0001; NT5E *p* < 0.001; CD34 *p* = 0.013; Supplementary Table S6).

## Discussion

DLBCL is a malignant neoplasm that can affect both pediatric and adult patients, with a better prognosis for pediatric patients^3^. Given the lack of knowledge about these differences, the aim of this study was to analyze gene expression characteristics to shed some light on the reasons for these differences. The gene expression analysis would allow the identification of several differentially expressed genes that may provide important information about the biological and clinical differences between pediatric and adult patients.

In the first approach, the 16 genes differentially expressed between pediatric and adult DLBCL NOS patients were involved in key processes related to cell differentiation and function, as well as the activation of oncogenic signaling pathways. In fact, the genes upregulated in pediatric patients, including both EBV + and EBV- cases, such as *NT5E*, *CD209*, *TET1* and *CD34*, are associated with B and T cell differentiation and function, as well as regulation of inflammation and antigen presentation. This may be probably related to its expression in a still immature immune system, undergoing differentiation and accumulation of immunological memory as an evolving feature of the adaptive immune response, compared to adult cases. This highlights age-related differences in immune function and tumor-host interactions. In addition, the overexpression in genes such as *IKBKB*,* ALK*,* MYC*,* CD69*,* CD19*,* NRP1*,* NOS3* and *PDGRFA*, associated with the activation of oncogenic signaling pathways, cell proliferation, angiogenesis and tumor progression, may suggest that pediatric DLBCL could be more aggressive than adult cases, even though survival is better in children^17^. Furthermore, overexpression of *MYC* and *ALK* was also demonstrated in two aggressive pediatric lymphomas, Burkitt and anaplastic, respectively^18^. Further studies in pediatric and adult patients will help determine whether MYC and/or ALK may be actionable targets for the treatment of relapsed or refractory DLBCL in children.

On the other hand, genes associated with chemotaxis and antigen presentation were found to be significantly downregulated in pediatric patients. These findings may suggest that pediatric patients may have a different immune response and antigenic presentation ability than adults. Furthermore, another important difference is the downregulation of the *PIM2* gene in children, which increased expression was associated with an aggressive clinical course in ABC DLBCL patients^19^. Therefore, its downregulation may counteract the aggressive behavior in pediatric patients.

KEGG pathway enrichment analysis was performed separately for the up- and downregulated genes, to increase the power to detect significant pathways. This analysis confirmed that, in pediatric DLBCL cases, the enriched pathways were involved with several cell proliferation-related pathways, reinforcing the idea of a more proliferative scenario, possibly related to an aggressive behavior in pediatric patients. In addition, given that this analysis also includes EBV + cases, most of them pediatric cases, an association with viral infection pathways, such as Epstein-Barr virus infection, was proved, as expected.

NK cells were associated with favorable clinical outcomes in DLBCL patients^20^, and a higher percentage of CD56dim was proved in DLBCL at diagnosis^21^, both in adult cases. Compared to adults, our pediatric DLBCL cases exhibited an increase in NKCD56dim cell-specific gene expression, providing an insight into this specific NK cell subpopulation and its differences in the tumor microenvironment between these two age groups. NKCD56dim cells rely on their cytotoxic activity to eliminate tumor cells^22^, suggesting that their increased presence in pediatric patients may be related to a more effective cytotoxic immune response against lymphoma in this age group, and this fact might explain, at least in part, the better outcome in this group, compared to adults^17^.

Given that EBV may influence the pathogenesis of DLBCL and the expression of cellular genes^14–16^, a second analysis excluding EBV + DLCBL cases was performed, in order to assess if EBV differentially influences gene expression in adults or pediatric DLBCL. This analysis showed that 10 new genes were downregulated in pediatric patients not associated with EBV compared to adults. Downregulated genes in the absence of EBV in children included genes associated with key immune regulators (*IL-10*) and immune checkpoint genes (*LAG3* and *CD274*), suggesting the impact of EBV infection in the modulation of immune response in pediatric lymphomas. This fact reveal the modulation of immune response by checkpoint inhibitors may be further study as actionable targets to treat pediatric EBV + DLBCL. In line with this, the influence of EBV in the regulation of the immune microenvironment was previously demonstrated by several groups, including ours^23–26^. Furthermore, this assumption was reinforced by KEGG analysis, which revealed that downregulated genes were shown to be associated with the downregulation of the PD1 checkpoint pathway in cancer, the T cell receptor signaling pathway, and, as expected, the interaction of viral proteins with cytokines and cytokine receptors, malaria and cytokine-cytokine receptor interaction. On the other hand, the upregulated genes showed a similar enrichment to the pathways affected in the whole DLBCL, NOS series.

In this second EBV- DLBCL group, a significant increase in gene expression of NKCD56dim cells, mast cells and dendritic cells was observed in pediatric cases. NKCD56dim cells play a key role in the control of primary infection in children^27^, in the prevention of viral-mediated lymphomagenesis^28^, and its decrease was observed in EBV + cHL patients compared to EBV- cHL patients^29^, and in posttransplant lymphoproliferative disorders^30^. Dendritic cell populations seem to play significant roles during primary EBV infection, in particular plasmacytoid dendritic cells (pDC). Moreover, EBV-induced lymphoma formation was observed after pDC depletion, and this was mediated by decreased NK cells^31^. Therefore, our results reinforce the notion that a decrease in DCs could be related to EBV-associated lymphomas in children, since they increase in EBV negative DLBCL cases. In contrast, a stronger immune activation in pediatric EBV- DLBCL patients characterized by NKCD56dim cells, mast cells and dendritic cells may indicate higher cytotoxic activity and a more pronounced inflammatory response compared to adult patients, counteracted by a regulatory response, as previously observed in pediatric lymphomas^14,32^. Identifying these differences in gene expression between pediatric and adult patients, particularly in EBV-negative cases, highlights the importance of considering both viral etiology and age in understanding and treating DLBCL.

Gene expression analysis and its relationship to survival in pediatric patients with DLBCL is scarce, based on the low incidence of DLBCL in children. Although definitive conclusions cannot be drawn due to the small sample size, this study showed associations between differentially significant gene expression and event-free survival in pediatric patients with DLBCL, in particular those expressing *MYC* above the median, and *NT5E* and *CD34* genes below the median, who had a lower event-free survival. In line with our findings, overexpression of c-MYC, an important oncogene playing a key role in Burkitt lymphoma, has previously been associated with poorer prognosis in patients with DLBCL^33^. Furthermore, in our series, *MYC* expression using the median as cut off value displayed a higher risk of event, and further studies in larger series would be required to prove its use as a predictive factor, and perhaps as an actionable target for differential treatment in children in the future. *NT5E* acts as an inhibitory immune checkpoint molecule, based on its ability to generate a free adenosine that inhibits cellular immune responses, thereby promoting the immune escape of tumor cells^34^. *NT5E*, a gene encoding the CD73 enzyme, has been found to be upregulated at multiple levels to support tumor growth in several subtypes of solid tumors, as well as in myeloma^34,35^, but in lymphoma its description is scant. CD73 expression in tumor cells, the protein encoded by *NT5E*, was described to be associated with poorer outcomes^36^. In addition, even though *NT5E* appears to support tumor growth at multiple levels, and shorter relapse free survival times in HNSCC, potentially due to overexpression of *NT5E*^34^, was described, its lower expression associated with worse survival in children with DLBCL may reflect a different behavior compared to adult patients. *CD34* gene expression has been extensively studied in several types of lymphoma. These include adult acute lymphoblastic leukemia (ALL) and childhood ALL. In adult ALL, CD34 positivity is associated with a poor prognosis, including older age, more extensive lymphoid organ involvement and higher serum LDH levels^37^. However, in pediatric ALL, contradicting results were observed, since CD34 expression was associated with favorable presentation and improved event-free survival^38^, like in our series of pediatric DLBCL, but also in pediatric T-cell ALL, CD34 expression is associated with poor survival^39^. These findings suggest that the role of *CD34* gene expression in lymphoma is complex and may vary depending on the specific type and patient population. Both *NT5E* and *CD34* genes presented a lower risk of death or relapse, also adjusted by age, sex and EBV infection, suggesting that, at least in this preliminary series, they may have significant consequences for lymphoma development, progression and response to treatment. However, as pointed out for *MYC* expression, definitive conclusions cannot be made due to the limited number of samples, and further studies in children are required to reveal their significance as predictive prognostic biomarkers.

In summary, this study provides a better understanding of the differences in the pathogenesis of DLBCL between children and adults, and the interaction with tumor microenvironment. Even though the small sample size in the clinicopathological analysis may affect the differences observed between groups, it could facilitate the discovery of new targets for innovative anti-lymphoma treatment strategies specifically for children. Thus, further validation studies in larger populations and in different geographical locations with different technical approaches should be conducted in order to verify our findings.

## Materials and methods

### Patients and samples

A total of 48 DLBCL patients, previously characterized^26,40^ were enrolled in this study, 26 pediatrics and 22 adults, collected retrospectively, based on the availability of sufficient material, from the archives at Pathology Division, at the Ricardo Gutierrez Children’s Hospital, and the Marie Curie Hospital in Buenos Aires, Argentina. Samples were obtained at diagnosis. FFPE biopsies from adults were obtained from lymph nodes, while 16 pediatric samples were extranodal. The age range was 2–73 years (total median: 16 years, pediatric median: 9 years, and adults: median 60 years). Institutional guidelines regarding human experimentation were followed, in accordance with the Helsinki Declaration of 1975. The Ricardo Gutierrez Children’s Hospital Ethics Committee (CEI) approved the study, and all the patients or patients’ guardians gave informed consent.

Of the 48 DLBCL cases, 11 were EBV + DLCBL NOS. Of those cases, 8 were pediatric and the 3 remaining were adults. Given the small number of cases, this study separated into two groups for further analysis, DLCBL, NOS and DLBCL excluding EBV + DLBCL NOS cases.

### RNA extraction

From formalin-fixed and paraffin-embedded (FFPE) biopsies, total RNA was isolated with RNeasy FFPE kit (Qiagen, Valencia, MA, EE. UU.), according to the manufacturer’s instructions, and quantified with NanoDrop 2000/2000 C (ThermoFisher Scientific, Waltham, MA, USA). The integrity and quality of the RNA were determined using TapeStation 4200 RNA ScreenTape kit (Agilent Technologies, Santa Clara, CA, USA), following the manufacturer’s instructions.

### nCounter gene-expression assay and pathway enrichment analysis

The Research Use Only version of the NanoString XT assay was used in conjunction with the nCounter Flex analysis system (NanoString Technologies, Seattle, WA, USA) to analyze gene expression. A custom 208-gene panel, previously characterized^9,10,26^, was used to assess gene expression. The gene panel included genes expressed by various components of the stroma and tumor cells in neoplastic disease, as well as numerous genes known to be therapeutic targets, and a set of 8 housekeeping genes to normalize gene expression levels.

The probes were hybridized to 200 ng of total RNA for 16 h at 65 °C. Excess was removed and immobilization of probe-transcription complexes was performed on a streptavidin-coated cartridge in the nCounter Preparation Station. Gene expression levels were normalized to housekeeping genes. Normalized counts were log2 transformed and agglomerative hierarchical clustering of gene expression was performed. Data were analyzed using nSolver analysis software 4.0 (NanoString Technologies).

An exhaustive search of the Genecard^41^ database for genes that were significantly up- or down-regulated was carried out to investigate the biological function of these genes.

The Kyoto Encyclopedia of Genes and Genomes (KEGG) is an online database with collected information of genomes, biological, chemicals and enzymatic pathways providing different types of functional links among genes or their products^42^. To understand the function of the differentially expressed genes, a pathway enrichment analysis based on KEGG of up and downregulated genes separately was performed, as previously suggested^43^, using ShinyGO 0.77 online tools (http://bioinformatics.sdstate.edu/go/*)*^44^. An FDR corrected p-value < 0.05 was considered statistically significant.

### Statistical analysis

For the analysis of the genes expressed by the cell types, Mann-Whitney U tests were performed to test for statistical differences in the median values between pediatric and adult patients. A p-value < 0.05 was considered statistically significant.

Since the focus of survival analysis was on pediatric patients, a total of 16 cases with available follow-up data were considered for survival analysis. The follow-up time was defined as the time from the date of diagnosis to either the last follow-up date or the occurrence of a specific event (death or relapse). Event-free survival (EFS) was measured from the date of diagnosis and first treatment to either the date of disease progression or discontinuation of treatment for any reason, or the censoring date if the patient was lost to follow-up or withdrew from the study. The group of patients with count gene expression values above and below the 50th percentile (median) was considered as cut off for separate patients in two groups for survival analysis.

The Kaplan-Meier method and log-rank test were used to estimate the groups’ survival curves and to determine the statistical significance of each marker.

Univariable Cox proportional hazard model was performed to estimate the association of the outcome variable with each gene expression. Gene expression with a significant p-value (*p* < 0.05) were tested in the multivariable model, adjusting by age (continuous with log transformation), sex (male/female), histologic subtype (GC/non-GC), and EBV status (negative/positive). The proportional hazard assumption was tested for each model. All statistical analyses were performed with survival and stat packages of R 4.2.2^45^.

## Electronic supplementary material

Below is the link to the electronic supplementary material.

Supplementary Material 1

## Data Availability

The datasets generated during and/or analyzed during the current study are available from the corresponding author on reasonable request.
