# Supplementary material for: Differential expression of proliferation and immune response genes between children and adults influences survival of diffuse large B cell lymphoma
Source: Sci Rep. 2025 Jun 3;15:19391. doi: 10.1038/s41598-025-04349-x (PMC12134336; doi:10.1038/s41598-025-04349-x)
Supplement: Supplementary file 1 — Supplementary Material 1 [file 41598_2025_4349_MOESM1_ESM.docx]

***Supplementary Table S1: Histological subtype for each age group****. The percent of patients in each histologic subgroup according to age group and sex are shown.*

| **Histological subtype** | **Pediatric** | | **Adult** | | **Total %** |
| --- | --- | --- | --- | --- | --- |
|  | **Male** | **Female** | **Male** | **Female** |  |
| **GC** | 8/21 | 5/21 | 5/21 | 3/21 | 21/48 (44) |
| **Non-GC** | 6/25 | 6/25 | 7/25 | 7/25 | 26/48 (54) |
| **ND** | 1/1 | 0/1 | 0/1 | 0/1 | 1/48 (2) |

***Supplementary Table S2: Differential expression of top 20 genes****: Pediatric vs. Adults DLCBL, NOS total cases.*

***Supplementary Table S3: Differential expression of top 20 genes****: Pediatric vs. Adults excluding EBV+ DLBCL cases.*

***Supplementary Fig. S1:*** ***Volcano blots plot****. The upregulated (red)/downregulated (blue) genes for Pediatrics vs Adults exclusively in EBV+ DLCBL cases.*


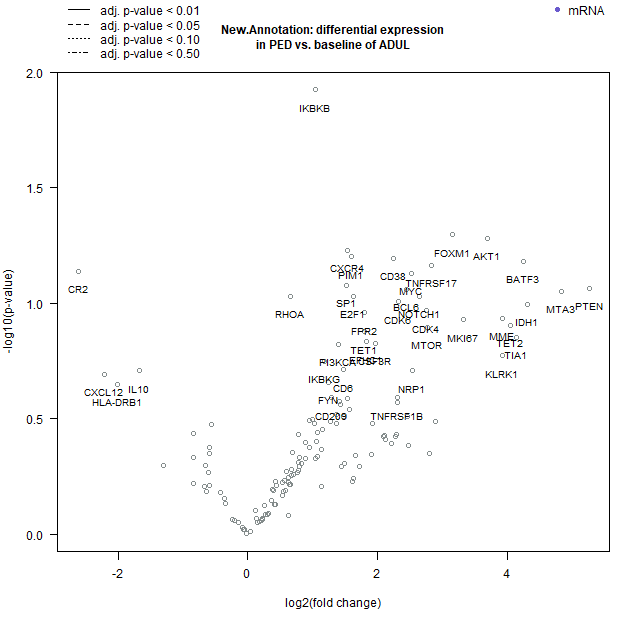


**Differential expression in Pediatrics vs. Baseline of Adults**

***Supplementary Table S4:*** ***KEGG Pathway enrichment analysis for differentially expressed genes for DLCBL, NOS cases (top 10 pathways with lower P-value)****. A) Upregulated genes B) Downregulated genes*

1. *Upregulated genes*

1. *Dowregulated genes*

***Supplementary Table S5***: ***Pathway enrichment analysis for differentially expressed genes for DLCBL without EBV+ DLCBL NOS cases (top 10 pathways with lower P-value).*** *A) Upregulated genes B) Downregulated genes*

*A) Upregulated genes*

*B) Downregulated genes*

***Supplementary Table S6:*** ***Univariable and multivariable analyses using Cox proportional-hazards models for survival in pediatric DLBCL****. Hazard ratios (HRs) from univariable analysis for covariates and genes expression. Multivariable analyses for genes expression that have shown significant results during univariable analyses. Error bars indicate the 95% confidence interval of the HR. Non-interpretable (NI) results are not shown.*

NI: non-interpretable. By "non-interpretable," we mean that the coefficient estimates for MYC lacked numerical stability or yielded implausibly wide confidence intervals.

*Multivariable analyses were limited to genes with significant or borderline-significant associations in univariable analyses to reduce model overfitting.

**Multivariable models adjusted for age, sex, EBV and histological subtype regardless of their univariable significance, due to their established clinical relevance (CITA si hay alguna).

***Given the small sample size (n=16), multivariate results should be interpreted cautiously.

**** *“NI” values reflect either unstable estimates or high p-values under the Cox multivariable model.
